# Critical B-lymphoid cell intrinsic role of endogenous MCL-1 in c-MYC-induced lymphomagenesis

**DOI:** 10.1038/cddis.2016.43

**Published:** 2016-03-10

**Authors:** S Grabow, G L Kelly, A R D Delbridge, P N Kelly, P Bouillet, J M Adams, A Strasser

**Affiliations:** 1 The Walter and Eliza Hall Institute of Medical Research, Melbourne, Victoria, Australia; 2 Department of Medical Biology, University of Melbourne, Melbourne, Victoria, Australia

## Abstract

Evasion of apoptosis is critical for tumorigenesis, and sustained survival of nascent neoplastic cells may depend upon the endogenous levels of pro-survival BCL-2 family members. Indeed, previous studies using gene-targeted mice revealed that BCL-XL, but surprisingly not BCL-2, is critical for the development of c-MYC-induced pre-B/B lymphomas. However, it remains unclear whether another pro-survival BCL-2 relative contributes to their development. MCL-1 is an intriguing candidate, because it is required for cell survival during early B-lymphocyte differentiation. It is expressed abnormally high in several types of human B-cell lymphomas and is implicated in their resistance to chemotherapy. To test the B-cell intrinsic requirement for endogenous MCL-1 in lymphoma development, we conditionally deleted *Mcl-1* in B-lymphoid cells of *Eμ-Myc* transgenic mice. We found that MCL-1 loss in early B-lymphoid progenitors delayed MYC-driven lymphomagenesis. Moreover, the lymphomas that arose when MCL-1 levels were diminished appeared to have been selected for reduced levels of BIM and/or increased levels of BCL-XL. These results underscore the importance of MCL-1 in lymphoma development and show that alterations in the levels of other cell death regulators can compensate for deficiencies in MCL-1 expression.

Apoptosis is a genetically programmed process for eliminating unwanted cells and is critical for normal development and tissue homeostasis in multi-cellular organisms.^1^ Defects in apoptosis are implicated in several disease states, particularly cancer^2^ and autoimmunity.^3^ Proteins of the BCL-2 family are major regulators of apoptosis.^4, 5^ The essential triggers are its BH3-only sub-family members (for example, BIM, PUMA and NOXA), which are activated transcriptionally and/or post-transcriptionally in response to diverse intracellular stresses.^6, 7^ The pro-apoptotic multi-BCL-2 homology (BH) domain proteins BAX, BAK (and possibly BOK^8^) have the essential role of permeabilizing the mitochondrial outer membrane, which constitutes the ‘point-of-no-return' in apoptosis signaling and unleashes the caspase cascade that mediates cell demolition.^4, 5, 9^ The pro-survival BCL-2 family members, including BCL-2, BCL-XL, MCL-1, BCL-W and A1/BFL1, counter the members of both these pro-apoptotic sub-families; they function in a cell type specific but frequently also overlapping manner. For example, MCL-1 is essential for early embryonic development^10^ and studies with conditional knockout mice revealed that it is critical for the survival of diverse cell types, including hematopoietic stem cells,^11^ immature as well as mature B- and T-lymphoid cells^12^ and certain myeloid cell populations.^13^

Many cancers display abnormalities in the levels of pro-survival and/or pro-apoptotic BCL-2 family members and evasion of apoptosis is widely thought to be essential to sustain the survival of nascent neoplastic cells and hence critical for tumorigenesis.^14, 15^ However, the mechanisms that protect cells undergoing neoplastic transformation from apoptosis remain incompletely understood.^2, 16^ Abnormalities in the BCL-2-governed apoptotic pathway or its regulators have been implicated in B-cell lymphoma development. For example, BCL-2 is overexpressed due to the t[14;18] chromosomal translocation in human follicular center B-cell lymphoma, whereas both alleles of *BIM* are frequently lost in mantle cell lymphoma.^17, 18, 19, 20^ Accordingly, transgenic overexpression of BCL-2 (or its relatives BCL-XL or MCL-1), or engineered loss of BIM, PUMA or BAX, can accelerate lymphomagenesis, particularly if cell cycle control is impaired, for example by enforced expression of c-MYC^21, 22, 23, 24, 25^ or v-Abl.^26^ Although lymphomas elicited by combined overexpression of c-MYC and BCL-2 are ‘addicted to' sustained BCL-2 overexpression for continued expansion,^27^ endogenous BCL-2 is dispensable for c-MYC-induced lymphomagenesis.^28^ In contrast, BCL-XL proved essential for the survival of both normal and pre-leukemic B cells undergoing neoplastic transformation and its loss greatly impaired lymphoma development in *Eμ-Myc* transgenic mice.^29^ Notably, the impaired tumor development could be overcome by concomitant loss of pro-apoptotic BIM.^30^

However, it is still unclear whether BCL-XL is the sole pro-survival BCL-2 family member required for MYC-induced pre-B/B-lymphoma development. MCL-1 is of particular interest. Increases in *MCL-1* gene copy number and concomitantly elevated MCL-1 protein are found in a substantial fraction of diverse cancer types.^31^ For a few cell lines derived from such cancers, *MCL-1* knockdown by RNA interference was shown to cause apoptosis, demonstrating that MCL-1 is critical for their sustained survival.^31^ Similarly, acute myeloid leukemia (AML) cells driven by enforced expression of c-MYC or the MLL-ENL and MLL-AF9 fusion onco-proteins and c-MYC- or BCR-ABL-driven pre-B/B lymphomas were rapidly killed upon inducible genetic deletion or blockade of MCL-1.^32, 33, 34, 35^

MCL-1 is critical for the survival of rapidly proliferating hematopoietic progenitors^36^ and non-transformed pro-B/pre-B cells,^12^ the cells thought to be the origin of *Eμ-Myc* lymphoma.^37, 38^ Therefore, we examined the role of MCL-1 in pre-B/B cell lymphoma development in *Eμ-Myc* transgenic mice by incorporating *CD19-Cre* or *Rag1-Cre* alleles to impose *Mcl-1* gene deletion exclusively in the B-lymphoid compartment. We report that there was marked selection against *Mcl-1* gene loss during c-MYC-driven lymphoma development and a delay in tumor onset. Moreover, the lymphomas that arose despite successful *Mcl-1*^*fl*^ recombination exhibited abnormally low levels of pro-apoptotic BIM and/or increased levels of pro-survival BCL-XL. These results show that MCL-1 is critical for c-MYC-driven pre-B/B-lymphoma development, and suggest that alterations in other core components of the apoptotic machinery can compensate for a reduction in MCL-1 levels.

## Results

### Impact of B-cell lineage-restricted deletion of Mcl-1 on MYC-driven lymphomagenesis

To explore the impact of B cell-restricted deletion of one or both allele(s) of *Mcl-1* on c-MYC-driven lymphoma development, we generated *Eμ-Myc* mice with one or both *Mcl-1* alleles flanked by *loxP* sites (hereafter called *Mcl-1*^*fl/+*^ or *Mcl-1*^*fl/fl*^, respectively). Some cohorts also expressed the Cre recombinase selectively either from the common lymphoid progenitor stage (CLP), using a *Rag-1-Cre* transgene, or from the late pro-B cell stage onwards, using a *CD19-Cre* transgene.^39^ In our *Mcl-1* gene-targeted mice, recombination of the *Mcl-1*^*fl*^ allele subjugates a human CD4 reporter transgene to the *Mcl-1* promoter/enhancer elements. Hence, human CD4 (hCD4) expression, which is readily detectable by flow cytometric analysis using fluorochrome-labeled anti-human-CD4 antibodies, serves as a reporter of *Mcl-1*^*fl*^ deletion.^33, 40, 41^

**Figure 1:**
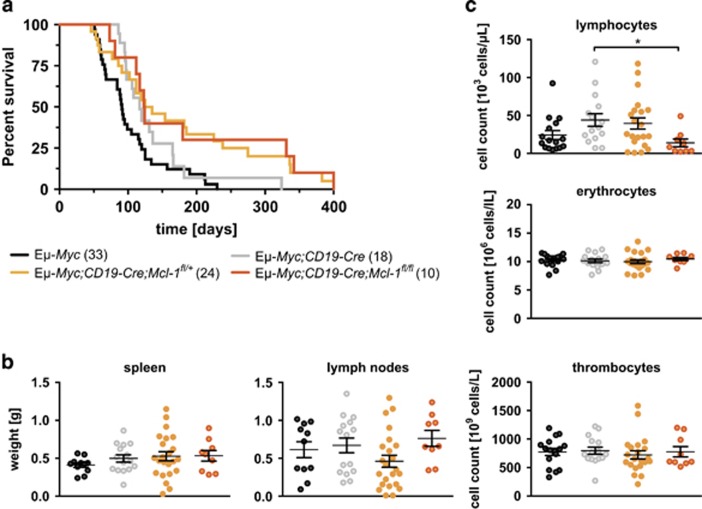
Minor impact of CD19-Cre-mediated loss of one or both alleles of *Mcl-1* in B-lymphoid cells on pre-B/B-cell lymphoma development in *Eμ-Myc* mice. (**a**) Kaplan–Meier survival curves comparing survival of *Eμ-Myc* (median 91 days), *Eμ-Myc*;*CD19-Cre* (117 days), *Eμ-Myc;CD19-Cre;Mcl-1*^*fl/+*^ (130 days) and *Eμ-Myc;CD19-Cre;Mcl-1*^*fl/fl*^ mice (123 days). *Eμ-Myc* *versus* *Eμ-Myc;CD19-Cre P*=0.069; *Eμ-Myc;CD19-Cre* *versus* *Eμ-Myc;CD19-Cre;Mcl-1*^*fl/+*^ *P*=0.16; *Eμ-Myc;CD19-Cre* *versus* *Eμ-Myc;CD19-Cre*;*Mcl-1*^*fl/fl*^ *P*=0.16). (**b**) Lymphoma burden in sick *Eμ-Myc*, *Eμ-Myc;CD19-Cre*, *Eμ-Myc;CD19-Cre;Mcl-1*^*fl/+*^and *Eμ-Myc;CD19-Cre;Mcl-1*^*fl/fl*^mice. No significant differences were observed, compared with sick *Eμ-Myc;CD19-Cre* mice, in the weights of the spleen or lymph nodes, respectively. (**c**) Peripheral blood analysis of sick, lymphoma-burdened compound mutant mice using an ADVIA counter. *Eμ-Myc;CD19-Cre* *versus* *Eμ-Myc;CD19-Cre;Mcl-1*^*fl/+*^ display no significant changes; *Eμ-Myc;CD19-Cre* *versus* *Eμ-Myc;CD19-Cre;Mcl-1*^*fl/fl*^: P*_lymphocytes_=0.0172)

We first compared the incidence and rate of pre-B/B-cell lymphoma development in *Eμ-Myc*, *Eμ-Myc;CD19-Cre*, *Eμ-Myc;CD19-Cre;Mcl-1*^*fl/+*^ and *Eμ-Myc;CD19-Cre;Mcl-1*^*fl/fl*^ mice (Figure 1a). The lymphoma-free survival of the control mice without *Mcl-1* deletion (*Eμ-Myc* and *Eμ-Myc;CD19-Cre*) was similar: median survivals of 91 days and 117 days, respectively (Mantle–Cox Log-rank test *P*=0.069, Figure 1a). With one or two floxed *Mcl-1* alleles, there was a modest (albeit not statistically significant) delay in lymphomagenesis compared with the *Eμ-Myc;CD19-Cre* animals: 130 and 123 days, respectively (*P*=0.16 for both).

Autopsy on the sick, lymphoma-burdened mice revealed that the *Eμ-Myc;CD19-Cre;Mcl-1*^*fl/fl*^ mice (P*=0.0172) had significantly less lymphoma cells in the blood than *Eμ-Myc;CD19-Cre* mice, but no such drop was found for the sick *Eμ-Myc;CD19-Cre;Mcl-1*^*fl/+*^mice. No significant differences between the genotypes appeared for spleen and lymph node weights (Figure 1b), or the numbers of erythrocytes and thrombocytes in the blood (Figure 1c).

### Selection against MCL-1 loss

**Figure 2:**
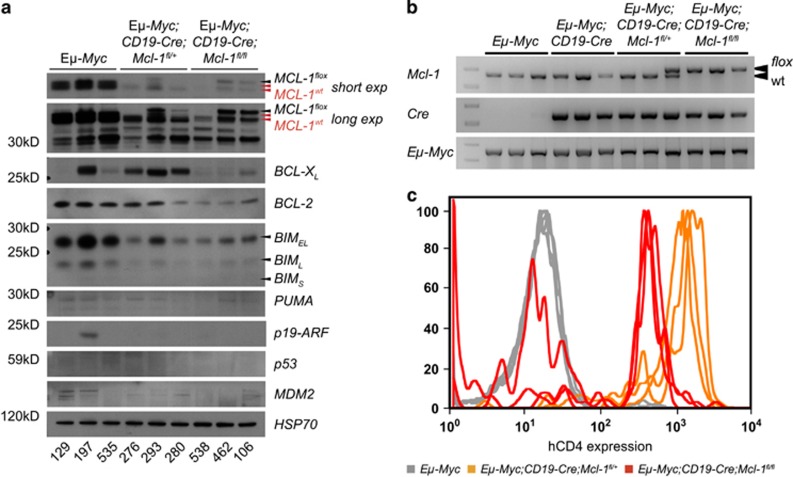
Expression of BCL-2 family members, p53, MDM2 and p19-ARF in lymphomas from *Eμ-Myc* mice with CD19-Cre-mediated deletion of *Mcl-1*. (**a**) The levels of the indicated proteins were determined by Western blot analysis in three lymphomas from each of the indicated genotypes. The protein from the *Mcl-1*^*fl*^ allele is slightly larger than the wt MCL-1 protein indicated by arrows.^53^ Probing for HSP70 was used as a loading control. (**b**) Genotype analysis of FACS-sorted B220^+^ pre-B/B-lymphoma cells from mice of the indicated genotypes, using primers that recognize both the *Mcl-1*^*wt*^ and *Mcl-1*^*fl*^ alleles, or detect the *Cre* recombinase transgene or the *Eμ-Myc* transgene. (**c**) Flow cytometric analysis of human CD4 reporter expression in lymphomas that arose in *Eμ-Myc* (negative control), *Eμ-Myc;CD19-Cre;Mcl-1*^*fl/+*^ or *Eμ-Myc;CD19-Cre;Mcl-1*^*fl/fl*^ mice

As constitutive or inducible loss of MCL-1 impairs the development as well as sustained expansion of many tumors,^33, 34, 41^ we tested whether the *Mcl-1*^*fl*^ allele(s) had been recombined in the lymphomas that arose in the *Eμ-Myc;CD19-Cre;Mcl-1*^*fl/+*^and *Eμ-Myc;CD19-Cre;Mcl-1*^*fl/fl*^ mice, or whether selection against *Mcl-1* gene loss had occurred during their malignant transformation. Western blot analysis revealed that many lymphomas from *Eμ-Myc;CD19-Cre;Mcl-1*^*fl/+*^(3/3 tested) and *Eμ-Myc;CD19-Cre;Mcl-1*^*fl/fl*^ mice (2/3 tested) retained MCL-1 expression, but its levels were significantly lower than in lymphomas from *Eμ-Myc* control mice (Figure 2a). The reduced MCL-1 protein expression appeared to be accompanied by a significant decrease in BIM protein expression in the *Eμ-Myc;CD19-Cre;Mcl-1*^*fl/+*^and *Eμ-Myc;CD19-Cre;Mcl-1*^*fl/fl*^ lymphomas tested. Also, BCL-XL appeared to be upregulated in the *Eμ-Myc;CD19-Cre;Mcl-1*^*fl/+*^ lymphomas, in which *Mcl-1*^*fl*^ deletion was efficient (Figure 2a). Compared with *Eμ-Myc;CD19-Cre* control lymphomas, BCL-2 protein expression was comparable in the *Eμ-Myc;CD19-Cre;Mcl-1*^*fl/+*^ lymphomas or, curiously, was lower in the *Eμ-Myc;CD19-Cre;Mcl-1*^*fl/fl*^ lymphomas that had retained their *Mcl-1*^*fl*^ alleles (Figure 2a). Consistent with the Western blot results, PCR analysis of FACS-sorted primary lymphoma cells confirmed that some of the lymphoma cells arising in *Eμ-Myc;CD19-Cre;Mcl-1*^*fl/+*^ and *Eμ-Myc;CD19-Cre;Mcl-1*^*fl/fl*^ mice had not excised or only partially excised their *Mcl-1*^*fl*^ alleles (Figure 2b).

Flow cytometric analysis for the human CD4 reporter confirmed that most tumors arising in *Eμ-Myc;CD19-Cre;Mcl-1*^*fl/+*^ mice had efficiently excised their *Mcl-1*^*fl*^ allele. However, only ~50–60% of the two floxed *Mcl-1* alleles had been recombined in the lymphomas from the *Eμ-Myc;CD19-Cre;Mcl-1*^*fl/fl*^ mice (Figure 2c). This suggests that the remaining wild-type (wt) *Mcl-1* allele in *Eμ-Myc;CD19-Cre;Mcl-1*^*fl/+*^ B-lymphoid cells is sufficient to sustain their survival during neoplastic transformation. In contrast, deletion of both *Mcl-1*^*fl*^ alleles must impose a stress from which lymphoma-initiating B-lymphoid progenitors are unable to recover. Thus, lymphomas that arise in *Eμ-Myc;CD19-Cre;Mcl-1*^*fl/fl*^ mice have potently selected against loss of both *Mcl-1*^*fl*^ alleles and the stress caused by loss of one *Mcl-1*^*fl*^ allele is partially relieved by adjustments in the levels of the BCL-XL and BIM proteins.

### Overexpression of c-MYC causes selection bias against deletion of Mcl-1^fl^ genes in pre-leukemic B-lymphoid cells

c-MYC promotes cell growth and cell proliferation, but under conditions of stress, such as nutrient or growth factor deprivation, high c-MYC levels predispose cells to undergo apoptosis.^42, 43, 44^ Pre-leukemic *Eμ*-*Myc* mice exhibit increased numbers of pre-B cells in their bone marrow, spleen, lymph nodes and blood, but these cells are not transformed and consequently do not form tumors when transplanted into congenic recipient mice.^37^

**Figure 3:**
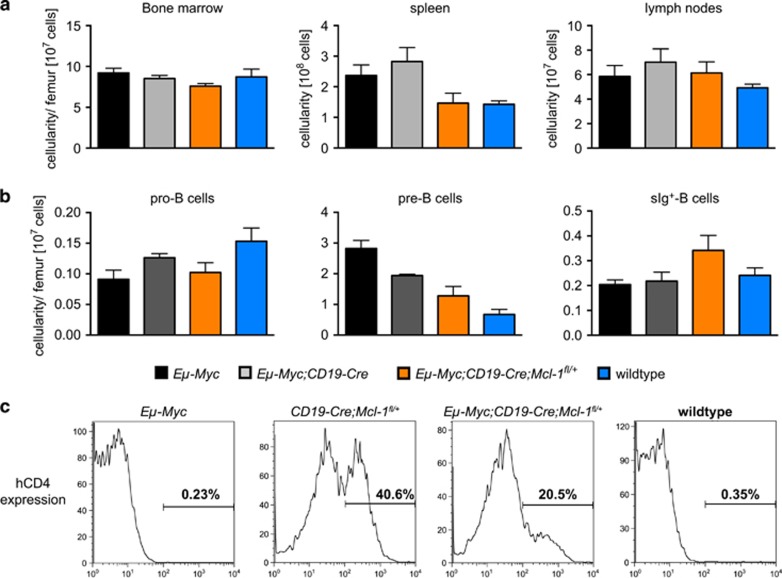
Impact of c-MYC overexpression on the deletion of *Mcl-1*^*fl*^ alleles in pre-leukemic B-lymphoid cells. (**a**) Total leukocyte numbers in the bone marrow, spleen and lymph nodes of 3–4-week-old pre-leukemic *Eμ-Myc*, *Eμ-Myc;CD19-Cre* and *Eμ-Myc;CD19-Cre;Mcl-1*^*fl/+*^ mice and wild-type (control) mice was determined by cell counting (*n*=3–5). (**b**) The total numbers of pro-B (B220^+^c-Kit^+^sIg^-^), pre-B (B220^+^c-Kit^-^sIg^-^) and sIg^+^-B cells (B220^+^c-Kit^-^sIg^+^) in the bone marrow of 3–4-week-old pre-leukemic wild-type (control), *Eμ-Myc*, *Eμ-Myc;CD19-Cre* and *Eμ-Myc;CD19-Cre;Mcl-1*^*fl/+*^ mice were determined by flow cytometric analysis. Comparison between *Eμ-Myc;CD19-Cre* and *Eμ-Myc;CD19-Cre;Mcl-1*^*fl/+*^mice: *P*_pro-B_=0.24; *P*_pre-B_=0.10; *P*_sIg+-B_=0.15 (*n*=3–5). (**c**) Expression of the human CD4 reporter on B-lymphoid cells (gated as B220^+^) from wild-type (control) and pre-leukemic *Eμ-Myc*, *Eμ-Myc;CD19-Cre* and *Eμ-Myc;CD19-Cre;Mcl-1*^*fl/+*^ mice was determined by flow cytometric analysis

Given that loss of one allele of *Mcl-1* suffices to potently induce cell death in malignant *Eμ-Myc* lymphomas,^35^ we hypothesized that loss of one *Mcl-1*^*fl*^ allele might also reduce the numbers of pre-leukemic pro-B, pre-B and/or sIg^+^-B cells (at 3–4 weeks of age) in *Eμ-Myc;CD19-Cre;Mcl-1*^*fl/+*^mice compared with *Eμ-Myc* and *Eμ-Myc;CD19-Cre* control animals. The total bone marrow and lymph node cellularities of pre-leukemic *Eμ-Myc, Eμ-Myc;CD19-Cre* and *Eμ-Myc;CD19-Cre;Mcl-1*^*fl/+*^ mice were comparable to each other and to wt mice, but there was a notable increase, although not statistically significant, in the overall leukocyte numbers in the spleens of *Eμ-Myc* and *Eμ-Myc;CD19-Cre* mice compared with the *Eμ-Myc;CD19-Cre;Mcl-1*^*fl/+*^ animals and the wt controls (Figure 3a). As reported,^37^ *Eμ-Myc* mice had more pre-B cells in their bone marrow than wt controls (Figure 3b). Interestingly, *Eμ-Myc;CD19-Cre* mice had significantly fewer pre-B cells than *Eμ-Myc* mice (Figure 3b; P*=0.0452), suggesting that the Cre recombinase imposes a cytotoxic stress on these cells. *Eμ-Myc;CD19-Cre;Mcl-1*^*fl/+*^ animals had even fewer pre-leukemic pre-B cells than the *Eμ-Myc;CD19-Cre* animals, but this difference was not statistically significant (*P*=0.10; Figure 3b), although the difference to the *Eμ-Myc* mice was significant. There were no significant differences in the numbers of pro-B cells or sIg^+^-B cells in the bone marrow between mice of any of the genotypes examined (Figure 3b).

As some lymphomas that arose in *Eμ-Myc;CD19-Cre;Mcl-1*^*fl/+*^ mice had been selected for retention of their *Mcl-1*^*fl*^ allele, we hypothesized that there may be potent selection against loss of the *Mcl-1*^*fl*^ allele already in the pre-leukemic state. To examine this, we stained spleen cells from pre-leukemic *Eμ-Myc;CD19-Cre;Mcl-1*^*fl/+*^ mice as well as those from *Eμ-Myc*, *CD19-Cre;Mcl-1*^*fl/+*^ and wt animals with antibodies against B220 (B-cell marker) and hCD4 (reporter for *Mcl-1*^*fl*^ deletion; Figure 3c). As expected, the B-lymphoid cells from *Eμ-Myc* and wt mice did not express hCD4. The B-lymphoid cells from *CD19-Cre;Mcl-1*^*fl/+*^mice were composed of two distinct populations, one negative (~60%) and the other positive (~40%) for hCD4 (Figure 3c). This demonstrates that some B-lymphoid cells in these animals were able to delete the *Mcl-1*^*fl*^ allele, although the efficiency was not very high. This is consistent with the previously reported^45^ relatively poor recombination efficiency of the *CD19-Cre* deletion strain that we used in our experiments. Interestingly, in the *Eμ-Myc;CD19-Cre;Mcl-1*^*fl/+*^ mice only ~20% of the pre-leukemic B-lymphoid cells were hCD4^+^ (Figure 3c).

These findings reveal that deregulated c-MYC expression exerts potent selection against loss of one *Mcl-1*^*fl*^ allele in B-lymphoid cells, whereas loss of one *Mcl-1* allele is more readily tolerated in normal B-lymphoid cells.

### Efficient deletion of *Mcl-1*^*fl*^ allele(s) in B-lymphoid progenitors using the *Rag-1-Cre* transgene substantially delays lymphomagenesis in *Eμ-Myc* mice

**Figure 4:**
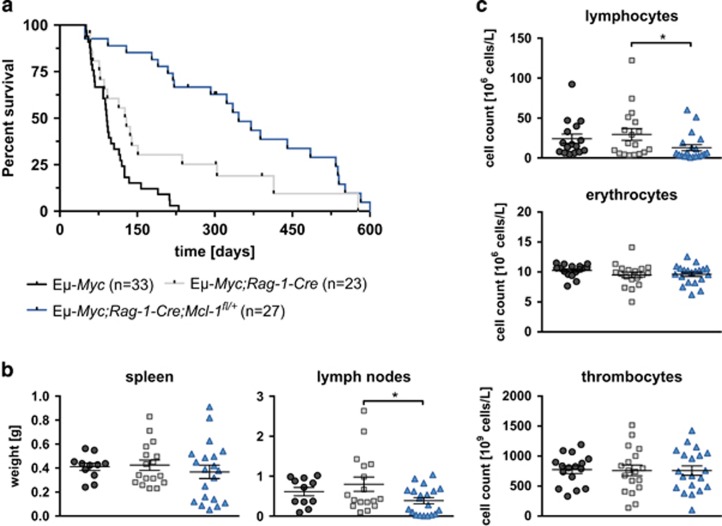
*Rag1-Cre*-mediated deletion of one allele of *Mcl-1* greatly delays lymphoma development in *Eμ-Myc* mice. (**a**) Kaplan–Meier animal survival curves comparing *Eμ-Myc*, *Eμ-Myc*;*Rag1-Cre* and *Eμ-Myc;Rag1-Cre;Mcl-1*^*fl/+*^ mice. *Eμ-Myc;Rag1-Cre* *versus* *Eμ-Myc;Rag1-Cre;Mcl-1*^*fl/+*^; Mantle–Cox Log-rank test P*=0.0030). Median onset of pre-B/B-cell lymphoma: *Eμ-Myc*: 91 days; *Eμ-Myc;Rag1-Cre*: 129 days, *Eμ-Myc;Rag1-Cre;Mcl-1*^*fl/+*^=346 days. (**b**) Lymphoma burden (spleen and lymph node weights) at autopsy in sick mice of indicated genotypes. A significant difference was observed in lymph node weights (P*=0.0307) between sick *Eμ-Myc;Rag1-Cre* and *Eμ-Myc;Rag1-Cre;Mcl-1*^*fl/+*^mice. (**c**) The numbers of lymphoid cells, erythrocytes and thrombocytes in the peripheral blood of lymphoma-burdened mice of the indicated genotypes were determined at autopsy using the ADVIA counter. *Eμ-MycRag-1-Cre* *versus* *Eμ-Myc;Rag-1-Cre;Mcl-1*^*fl/+*^: P*_lymph_=0.0464)

As *CD19-Cre*-mediated deletion of *Mcl-1*^*fl*^ alleles was rather inefficient, we wanted to test whether deleting *Mcl-1*^*fl*^ allele(s) more efficiently and at an earlier stage in B-cell development would have a greater impact in our lymphoma model. For this we employed the *Rag-1-Cre* knockin allele, which was reported to recombine floxed genes with very high efficiency at the CLP stage.^46, 47^ Lymphoma onset was slightly delayed in the *Eμ-Myc;Rag-1-Cre* mice compared with the *Eμ-Myc* control animals. Although this difference was not significant (*P*=0.06), this indicates that the *Rag-1-Cre* transgene exerts some toxicity on B-lymphoid cells undergoing neoplastic transformation. Remarkably, the median lymphoma-free survival of *Eμ-Myc;Rag-1-Cre;Mcl-1*^*fl/+*^ mice (346 days) was far longer than in control *Eμ-Myc* (91 days) and *Eμ-Myc;Rag-1-Cre* mice (129 days, P**=0.003, Figure 4a), clearly demonstrating the importance of MCL-1 in c-MYC-induced lymphomagenesis.

The lymphoma-burdened, sick *Eμ-Myc;Rag-1-Cre;Mcl-1*^*fl/+*^ mice showed significantly lower lymph node weights (*P=0.031) and lymphocyte numbers in the peripheral blood (*P=0.046) than sick *Eμ-Myc;Rag-1-Cre* mice (Figures 4b and c). No significant differences were found in the spleen weights or in the numbers of erythrocytes and thrombocytes in the blood.

**Figure 5:**
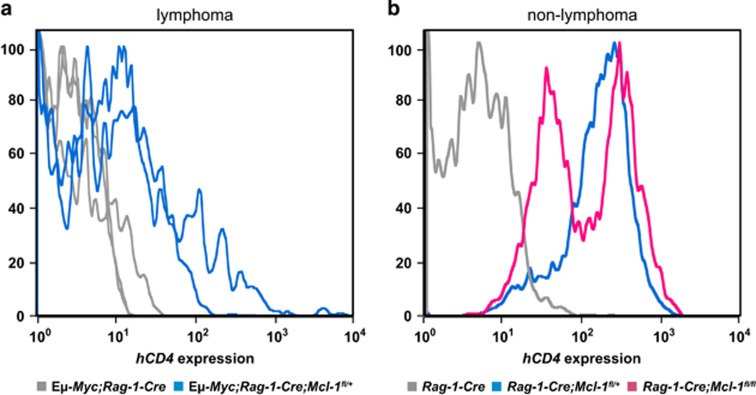
Lymphomas from *Eμ-Myc;Rag-1-Cre;Mcl-1*^*fl/+*^ mice are selected against loss of their *Mcl-1*^*fl*^ allele. (**a**) Flow cytometric analysis of hCD4 reporter expression (reflecting *Mcl-1*^*fl*^ recombination) on lymphoma cells from *Eμ-Myc* (control), *Eμ-Myc;Rag-1-Cre* and *Eμ-Myc;Rag-1-Cre;Mcl-1*^*fl/+*^ mice. (**b**) Flow cytometric analysis of hCD4 reporter expression on normal B-lymphoid cells (gated as B220^+^) from 3–4-week-old *Rag-1-Cre; Rag1-Cre;Mcl-1*^*fl/+*^ and *Rag1-Cre;Mcl-1*^*fl/fl*^ mice

The marked delay in lymphoma development seen in the *Eμ-Myc;Rag-1-Cre;Mcl-1*^*fl/+*^ mice suggested that *Rag-1-Cre* was considerably more efficient in *Mcl-1*^*fl*^ deletion than *CD19-Cre*. To test this hypothesis, we analyzed lymphoma cells from *Eμ-Myc;Rag-1-Cre* and *Eμ-Myc;Rag-1-Cre;Mcl-1*^*fl/+*^ mice for hCD4 expression (Figure 5a). Strikingly, the selection against cells expressing the hCD4 reporter (i.e. selection against cells that had deleted the *Mcl-1*^*fl*^ allele) was clearly more potent in *Eμ-Myc;Rag-1-Cre;Mcl-1*^*fl/+*^ lymphoma cells than in those from the *Eμ-Myc;CD19-Cre;Mcl-1*^*fl/+*^ mice (compare data in Figures 2c and 5a). In the absence of oncogenic stress, *Rag-1-Cre;Mcl-1*^*fl/+*^mice efficiently deleted one *Mcl-1* allele in B-lymphoid cells, but interestingly, there was potent selection against loss of both *Mcl-1* alleles even without c-MYC overexpression (Figure 5b). These results reveal that non-transformed B-lymphoid cells can tolerate loss of one but not loss of both *Mcl-1* alleles, whereas cells with deregulated c-MYC expression (both pre-leukemic cells undergoing transformation as well as malignant lymphomas) cannot tolerate even loss of a single allele. Unfortunately we were unable to generate *Eμ-Myc;Rag-1-Cre;Mcl-1*^*fl/fl*^ mice due to issues with infertility.

Collectively, these data show that *Mcl-1* is essential for the survival of MYC overexpressing pre-leukemic B-lymphoid cells undergoing neoplastic transformation. Therefore, B-lymphoid-restricted loss of one allele of *Mcl-1* can substantially delay pre-B/B-lymphoma development in *Eμ-Myc* mice.

## Discussion

Evasion of cell death is considered an essential requirement for the development of cancer.^2^ Impaired apoptosis in cancer cells (particularly in hematological malignancies) often results from deregulated expression of pro-survival or pro-apoptotic members of the BCL-2 protein family.^48^ In cells undergoing neoplastic transformation, apoptosis can be triggered by stress conditions induced by newly acquired oncogenic mutations (e.g. deregulated c-MYC expression) or by limiting availability of nutrients or growth factors from the tumor micro-environment. Regardless of the trigger that activates apoptosis signaling, evasion of cell death is essential for a population of nascent neoplastic cells to expand and sub-clones to acquire additional oncogenic lesions that cooperate with the initiating oncogenic mutation(s) to promote emergence of malignant cells.^16^

Although BCL-2 overexpression greatly accelerates lymphomagenesis in *Eμ*-*Myc* transgenic mice,^25^ endogenous BCL-2 is dispensable for MYC-driven lymphoma development.^28^ In contrast, BCL-XL was found to be essential for the survival of both normal as well as c-MYC overexpressing B-cell progenitors and its loss therefore inhibited lymphoma development in *Eμ*-*Myc* mice.^29^ Here we show that MCL-1 is also critical for c-MYC-driven lymphoma development.

We employed two Cre transgenic strains to delete *Mcl-1* either at the late pro-B cell (*CD19-Cre*^39^) or the CLP stage (*Rag-1-Cre*^46, 47^). Surprisingly, we found that lymphoma development in the *Eμ*-*Myc;CD19-Cre;Mcl-1*^*fl/+*^ and *Eμ*-*Myc;CD19-Cre;Mcl-1*^*fl/fl*^ mice was only slightly slower than in the control *Eμ*-*Myc* and *Eμ*-*Myc;CD19-Cre* mice. The difference to the *Eμ*-*Myc* mice was statistically significant but the difference to the *Eμ*-*Myc;CD19-Cre* mice was not, probably because constitutive Cre activity imposes a slight toxicity in B-lymphoid cells, as previously observed in other cell types.^49^ Interestingly, in young, pre-leukemic *Eμ-Myc;CD19-Cre;Mcl-1*^*fl/+*^ mice considerably fewer B-lymphoid cells had deleted their *Mcl-1*^*fl*^ allele (detected as human CD4^+^) than in the *CD19-Cre;Mcl-1*^*fl/+*^ animals. This demonstrates that deregulated c-MYC expression renders nascent neoplastic cells exquisitely dependent on an adequate MCL-1 protein level (i.e., provided by both *Mcl-1* alleles) for their survival. This selection against pre-leukemic B-lymphoid cells that had deleted their *Mcl-1*^*fl*^ allele(s) explains why some pre-B/B lymphomas arising in *Eμ*-*Myc;CD19-Cre;Mcl-1*^*fl/+*^ and *Eμ*-*Myc;CD19-Cre;Mcl-1*^*fl/fl*^ mice had been selected against loss of their *Mcl-1*^*fl*^ allele(s). Thus, cells retaining their full MCL-1 complement had an advantage in progressing through further steps of neoplastic transformation. Moreover, lymphomas that arose in *Eμ*-*Myc;CD19-Cre;Mcl-1*^*fl/+*^ and *Eμ*-*Myc;CD19-Cre;Mcl-1*^*fl/fl*^ mice despite loss of one *Mcl-1* allele appeared to have undergone selection for upregulation of BCL-XL and/or a reduction in pro-apoptotic BIM. This in turn suggests that keeping BIM in check constitutes a major function for MCL-1 in B-lymphoid cells undergoing transformation.

Lymphoma-free survival was extended to a much greater extent in *Eμ*-*Myc;Rag-1-Cre;Mcl-1*^*fl/+*^ mice compared with the *Eμ*-*Myc;CD19-Cre;Mcl-1*^*fl/+*^ and *Eμ*-*Myc;CD19-Cre;Mcl-1*^*fl/fl*^ animals. This may indicate that loss of one *Mcl-1* allele at the earlier CLP stage of lymphoid cell development (i.e., when *Rag1-Cre* but not *CD19-Cre* is expressed) is more efficient in killing incipient neoplastic cells and therefore more efficient in delaying lymphoma development compared with *Mcl-1*^*fl*^ deletion at the later pro-B-cell stage (when *CD19-Cre* expression commences). Alternatively, the *Rag1-Cre* transgene may simply be more efficient than the *CD19-Cre* transgene; the latter would therefore more readily allow escape of B-lymphoid cells that had failed to excise *Mcl-1*^*fl*^.

In conclusion, our findings demonstrate that MCL-1 is critical for the survival of c-MYC overexpressing lymphoma-initiating cells and hence for development of lymphoma. MCL-1 appears to be more important than BCL-XL because loss of one *Mcl-1* allele substantially delayed lymphoma development in *Eμ*-*Myc;Rag-1-Cre;Mcl-1*^*fl/+*^ mice, whereas loss of one *Bclx* allele had only minor impact.^29, 30^ Loss of BIM-restored lymphoma development in mice with an *Eμ*-*Myc;Bclx*^*−/−*^ lymphoid system and many pre-B/B lymphomas that arose in *Eμ*-*Myc;CD19-Cre;Mcl-1*^*fl/+*^ or *Eμ*-*Myc;CD19-Cre;Mcl-1*^*fl/fl*^ mice despite loss of one *Mcl-1* allele appeared to have undergone selection for low levels of BIM. This suggests that BIM is the critical pro-apoptotic BH3-only protein activated in response to oncogenic stress to kill *Eμ*-*Myc* pre-leukemic B-lymphoid cells to suppress progression to malignant lymphoma. These results and the observation that loss of even a single allele of *Mcl-1* efficiently kills malignant *Eμ*-*Myc* lymphoma cells^41^ provide further impetus to develop MCL-1 specific inhibitors (e.g. BH3 mimetics) for cancer therapy.^50, 51^

## Materials and methods

### Experimental mice

All experiments with mice were conducted according to the guidelines of The Walter and Eliza Hall Institute of Medical Research Animal Ethics Committee. *Eμ-Myc* transgenic mice (generated on a mixed C57BL/6xSJL background and then backcrossed for >30 generations onto a C57BL/6 background) expressing the *c-Myc* transgene under control of the immunoglobulin heavy chain gene enhancer *Eμ* have been previously reported.^52^ The *Mcl-1*^*fl*^ mice were generated on a C57BL/6 background using C57BL/6-derived ES cells.^40^ The *Rag-1-Cre*^*Ki/+*^^46^ and *CD19-Cre*^*Ki/+*^^39^ mice were generated on a mixed C57BL/6x129SV genetic background using 129SV-derived ES cells and then backcrossed onto a C57BL/6 background for >20 generations before commencement of our studies.

### Genotyping

Genotyping was performed as previously reported.^36^ Oligonucleotide sequences for genotyping of these alleles will be provided on request.

### Analysis of lymphoma-burdened mice

*Eμ-Myc* transgenic mice were examined daily by animal technicians for signs of malignant disease. Mice were sacrificed when declared unwell by the animal technicians. Signs of disease included splenomegaly, lymphadenopathy, hind-limb paralysis, hunched stature, weight loss and labored breathing (indicative of lymphoma growth in the thymus). Sick mice were euthanized, tissues removed, weighed and then used for flow cytometric as well as histological analyses and tissue culture.

### Western blot analysis

Cells were lysed in RIPA buffer supplemented with a protease inhibitor cocktail (Roche, Basel, Switzerland). Protein lysates (30 *μ*g protein) mixed with 4x Laemmli buffer were loaded onto a 10% Bis/Tris gel (Life Technologies, Scoresby, VIC, Australia) and electrophoresis was conducted according to the manufacturer's instructions. Proteins were transferred onto nitrocellulose membranes using the iBlot system (Life Technologies, Scoresby, VIC, Australia). Nitrocellulose membranes were blocked for 2 h using 5% skim milk powder dissolved in phosphate-buffered saline supplemented with 0.5% Tween-20. Western blots were probed with the following monoclonal or polyclonal antibodies: rabbit anti-mouse MCL-1 (19C4-15), hamster anti-mouse BCL-2 (3F11), mouse anti-mouse BCL-XL (BD Pharmingen, BD BioSciences, San Jose, CA, USA; 2F12), rabbit anti-mouse BIM (Stressgen, 9292), rabbit anti-mouse PUMA (Ab-27669, Abcam, Melbourne, Victoria, Australia), mouse anti-HSP70 (R Anderson, Peter McCallum Cancer Centre; loading control), rabbit anti-mouse p53 (Leica Biosystems, Mount Waverley, Victoria, Australia; CM5) and rat anti-mouse p19-ARF (Rockland Immunochemicals, Pottstown, PA, USA; 5.C3.1), overnight at 4 °C. Blots were washed three times in phosphate-buffered saline supplemented with 0.5% Tween-20. The blots were then incubated for 1 h at room temperature with secondary HRP-conjugated antibodies against mouse, rat, hamster or rabbit IgG and again washed before exposure to the Amersham ECL reaction and developing on an autoradiograph Hyperfilm (GE Healthcare, Parramatta, NSW, Australia).

### Lymphoma and pre-leukemic analysis by flow cytometry

Lymphoid organs were harvested from lymphoma-burdened mice and single-cell suspensions prepared using forceps. Cells (5 × 10^6^) were resuspended in buffered saline supplemented with 10% FCS and 2% normal rat serum and stained for 30 min at 4 °C with rat monoclonal antibodies to B220 (RA3-6B2, The Walter and Eliza Hall Institute (WEHI)), cKIT (ACK4, WEHI), IgM (5.1, WEHI) and IgD (11-26, WEHI; all produced and conjugated with fluorochromes in our laboratory) and mouse monoclonal antibody to human CD4 (BD Pharmingen #555347, RPA-T4).

### Statistical analysis

Kaplan–Meier mouse survival curves were generated and analyzed with GraphPad Prism (GraphPad Software Inc, La Jolla, CA, USA). Mouse cohorts were compared using the log-rank Mantel–Cox test. *P*-values of <0.05 were considered significant. *In vitro* cell survival, blood cell counts, organ weights and RNA levels were plotted and analyzed with GraphPad Prism using two-tailed student's *t*-test comparing two groups with each other. Error bars are presented as standard error of mean (±s.e.m.).

## Abbreviations

BH3: BCL-2 Homology domain 3
BIM: BCL-2-interacting mediator of cell death
PUMA: p53 upregulated modulator of apoptosis
Noxa: phorbol-12-myr- istate-13-acetate-induced protein 1
BAK: BCL-2 antagonist/killer-1
BAX: BCL-2-associated X protein
BOK: BCL-2 related ovarian killer
BCL-2: B-cell lymphoma 2
BCL-XL: BCL-2-like 1 extra long
MCL-1: myeloid cell leukemia-1
BCL-W: BCL-2-like 2
A1/BFL-1: BCL-2-related protein A1
Abbreviations: Bcl-2 related gene in the fetal liver
MLL-ENL: mixed lineage leukemia-eleven-nineteen leukemia
MLL-AF9: ALL1 fused gene from chromosome 9
Rag-1: recombination activating gene 1
CD4: cluster of differentiation 4
Cre: cyclization recombination
CD10: cluster of differentiation 19
PCR: polymerase chain reaction
FACS: fluorescence-activated cell sorting

## References

[bib1] Hotchkiss RS, Strasser A, McDunn JE, Swanson PE. Cell death. New Engl J Med 2009; 361: 1570–1583. 19828534 10.1056/NEJMra0901217 PMC3760419

[bib2] Hanahan D, Weinberg RA. Hallmarks of cancer: the next generation. Cell 2011; 144: 646–674. 21376230 10.1016/j.cell.2011.02.013

[bib3] Strasser A, Harris AW, Cory S. *Bcl-2* transgene inhibits T cell death and perturbs thymic self-censorship. Cell 1991; 67: 889–899. 1959134 10.1016/0092-8674(91)90362-3

[bib4] Youle RJ, Strasser A. The BCL-2 protein family: opposing activities that mediate cell death. Nat Rev Mol Cell Biol 2008; 9: 47–59. 18097445 10.1038/nrm2308

[bib5] Czabotar PE, Lessene G, Strasser A, Adams JM. Control of apoptosis by the BCL-2 protein family: implications for physiology and therapy. Nat Rev Mol Cell Biol 2014; 15: 49–63. 24355989 10.1038/nrm3722

[bib6] Huang DCS, Strasser A. BH3-only proteins – essential initiators of apoptotic cell death. Cell 2000; 103: 839–842. 11136969 10.1016/s0092-8674(00)00187-2

[bib7] Puthalakath H, Strasser A. Keeping killers on a tight leash: transcriptional and post-translational control of the pro-apoptotic activity of BH3-only proteins. Cell Death Differ 2002; 9: 505–512. 11973609 10.1038/sj.cdd.4400998

[bib8] Ke F, Voss A, Kerr JB, O'Reilly LA, Tai L, Echeverry N et al. BCL-2 family member BOK is widely expressed but its loss has only minimal impact in mice. Cell Death Differ 2012; 19: 915–925. 22281706 10.1038/cdd.2011.210 PMC3354060

[bib9] Chipuk JE, Green DR. How do BCL-2 proteins induce mitochondrial outer membrane permeabilization? Trends Cell Biol 2008; 18: 157–164. 18314333 10.1016/j.tcb.2008.01.007 PMC3242477

[bib10] Rinkenberger JL, Horning S, Klocke B, Roth K, Korsmeyer SJ. Mcl-1 deficiency results in peri-implantation embryonic lethality. Genes Dev 2000; 14: 23–27. 10640272 PMC316347

[bib11] Opferman J, Iwasaki H, Ong CC, Suh H, Mizuno S, Akashi K et al. Obligate role of anti-apoptotic MCL-1 in the survival of hematopoietic stem cells. Science 2005; 307: 1101–1104. 15718471 10.1126/science.1106114

[bib12] Opferman JT, Letai A, Beard C, Sorcinelli MD, Ong CC, Korsmeyer SJ. Development and maintenance of B and T lymphocytes requires antiapoptotic MCL-1. Nature 2003; 426: 671–676. 14668867 10.1038/nature02067

[bib13] Dzhagalov I St, John A, He YW. The antiapoptotic protein Mcl-1 is essential for the survival of neutrophils but not macrophages. Blood 2007; 109: 1620–1626. 17062731 10.1182/blood-2006-03-013771 PMC1794052

[bib14] Letai AG. Diagnosing and exploiting cancer's addiction to blocks in apoptosis. Nat Rev Cancer 2008; 8: 121–132. 18202696 10.1038/nrc2297

[bib15] Adams JM, Cory S. The Bcl-2 apoptotic switch in cancer development and therapy. Oncogene 2007; 26: 1324–1337. 17322918 10.1038/sj.onc.1210220 PMC2930981

[bib16] Adams JM, Cory S. Bcl-2-regulated apoptosis: mechanism and therapeutic potential. Curr Opin Immunol 2007; 19: 488–496. 17629468 10.1016/j.coi.2007.05.004 PMC2754308

[bib17] Strasser A, Harris AW, Vaux DL, Webb E, Bath ML, Adams JM et al. Abnormalities of the immune system induced by dysregulated *bcl*-2 expression in transgenic mice. Curr Top Microbiol Immunol 1990; 166: 175–181. 2073796 10.1007/978-3-642-75889-8_22

[bib18] Tsujimoto Y, Cossman J, Jaffe E, Croce CM. Involvement of the *bcl*-2 gene in human follicular lymphoma. Science 1985; 228: 1440–1443. 3874430 10.1126/science.3874430

[bib19] Tsujimoto Y, Gorham J, Cossman J, Jaffe E, Croce CM. The t(14;18) chromosome translocations involved in B-cell neoplasms result from mistakes in VDJ joining. Science 1985; 229: 1390–1393. 3929382 10.1126/science.3929382

[bib20] Tagawa H, Karnan S, Suzuki R, Matsuo K, Zhang X, Ota A et al. Genome-wide array-based CGH for mantle cell lymphoma: identification of homozygous deletions of the proapoptotic gene BIM. Oncogene 2005; 24: 1348–1358. 15608680 10.1038/sj.onc.1208300

[bib21] Egle A, Harris AW, Bath ML, O'Reilly L, Cory S. VavP-*Bcl2* transgenic mice develop follicular lymphoma preceded by germinal center hyperplasia. Blood 2004; 103: 2276–2283. 14630790 10.1182/blood-2003-07-2469

[bib22] Egle A, Harris AW, Bouillet P, Cory S. Bim is a suppressor of Myc-induced mouse B cell leukemia. Proc Natl Acad Sci USA 2004; 101: 6164–6169. 15079075 10.1073/pnas.0401471101 PMC395940

[bib23] Hemann MT, Zilfou JT, Zhao Z, Burgess DJ, Hannon GJ, Lowe SW. Suppression of tumorigenesis by the p53 target PUMA. Proc Natl Acad Sci USA 2004; 101: 9333–9338. 15192153 10.1073/pnas.0403286101 PMC438977

[bib24] Michalak EM, Jansen ES, Happo L, Cragg MS, Tai L, Smyth GK et al. Puma and to a lesser extent Noxa are suppressors of Myc-induced lymphomagenesis. Cell Death Differ 2009; 16: 684–696. 19148184 10.1038/cdd.2008.195 PMC2743939

[bib25] Strasser A, Harris AW, Bath ML, Cory S. Novel primitive lymphoid tumours induced in transgenic mice by cooperation between *myc* and *bcl*-2. Nature 1990; 348: 331–333. 2250704 10.1038/348331a0

[bib26] Vandenberg CJ, Cory S. ABT-199, a new Bcl-2-specific BH3 mimetic, has in vivo efficacy against aggressive Myc-driven mouse lymphomas without provoking thrombocytopenia. Blood 2013; 121: 2285–2288. 23341542 10.1182/blood-2013-01-475855 PMC3606065

[bib27] Letai A, Sorcinelli MD, Beard C, Korsmeyer SJ. Antiapoptotic BCL-2 is required for maintenance of a model leukemia. Cancer Cell 2004; 6: 241–249. 15380515 10.1016/j.ccr.2004.07.011

[bib28] Kelly PN, Puthalakath H, Adams JM, Strasser A. Endogenous bcl-2 is not required for the development of E*μ*-myc-induced B-cell lymphoma. Blood 2007; 109: 4907–4913. 17317859 10.1182/blood-2006-10-051847 PMC1885522

[bib29] Kelly PN, Grabow S, Delbridge ARD, Strasser A, Adams JM. Endogenous Bcl-xL is essential for Myc-driven lymphomagenesis in mice. Blood 2011; 118: 6380–6386. 21998213 10.1182/blood-2011-07-367672 PMC3236120

[bib30] Delbridge AR, Grabow S, Bouillet P, Adams JM, Strasser A. Functional antagonism between pro-apoptotic BIM and anti-apoptotic BCL-XL in MYC-induced lymphomagenesis. Oncogene 2015; 34: 1872–1876. 24858047 10.1038/onc.2014.132

[bib31] Beroukhim R, Mermel CH, Porter D, Wei G, Raychaudhuri S, Donovan J et al. The landscape of somatic copy-number alteration across human cancers. Nature 2010; 463: 899–905. 20164920 10.1038/nature08822 PMC2826709

[bib32] Xiang Z, Luo H, Payton JE, Cain J, Ley TJ, Opferman JT et al. Mcl1 haploinsufficiency protects mice from Myc-induced acute myeloid leukemia. J Clin Invest 2010; 120: 2109–2118. 20484815 10.1172/JCI39964 PMC2877934

[bib33] Glaser SP, Lee EF, Trounson E, Bouillet P, Wei A, Fairlie WD et al. Anti-apoptotic Mcl-1 is essential for the development and sustained growth of acute myeloid leukemia. Genes Dev 2012; 26: 120–125. 22279045 10.1101/gad.182980.111 PMC3273836

[bib34] Koss B, Morrison J, Perciavalle RM, Singh H, Rehg JE, Williams RT et al. Requirement for antiapoptotic MCL-1 in the survival of BCR-ABL B-lineage acute lymphoblastic leukemia. Blood 2013; 122: 1587–1598. 23881917 10.1182/blood-2012-06-440230 PMC3757371

[bib35] Kelly GL, Grabow S, Glaser SP, Fitzsimmons L, Aubrey BJ, Okamoto T et al. Targeting of MCL-1 kills MYC-driven mouse and human lymphomas even when they bear mutations in p53. Genes Dev 2014; 28: 58–70. 24395247 10.1101/gad.232009.113 PMC3894413

[bib36] Delbridge A, Opferman JT, Grabow S, Strasser A. Pro-survival MCL-1 and pro-apoptotic PUMA govern stem/progenitor cell survival during emergency hematopoiesis. Blood 2015; 125: 3273–3280. 25847014 10.1182/blood-2015-01-621250 PMC4440882

[bib37] Langdon WY, Harris AW, Cory S, Adams JM. The c-*myc* oncogene perturbs B lymphocyte development in E*μ*-*myc* transgenic mice. Cell 1986; 47: 11–18. 3093082 10.1016/0092-8674(86)90361-2

[bib38] Harris AW, Pinkert CA, Crawford M, Langdon WY, Brinster RL, Adams JM. The E*μ*-*myc* transgenic mouse: a model for high-incidence spontaneous lymphoma and leukemia of early B cells. J Exp Med 1988; 167: 353–371. 3258007 10.1084/jem.167.2.353 PMC2188841

[bib39] Rickert RC, Roes J, Rajewsky K. B lymphocyte-specific, Cre-mediated mutagenesis in mice. Nucleic Acids Res 1997; 25: 1317–1318. 9092650 10.1093/nar/25.6.1317 PMC146582

[bib40] Vikstrom I, Carotta S, Lüthje K, Peperzak V, Jost PJ, Glaser S et al. Mcl-1 is essential for germinal center formation and B cell memory. Science 2010; 330: 1095–1099. 20929728 10.1126/science.1191793 PMC2991396

[bib41] Kelly GL, Grabow S, Glaser SP, Fitzsimmons L, Aubrey BJ, Okamoto T et al. Targeting of MCL-1 kills MYC-driven mouse and human lymphoma cells even when they bear mutations in p53. Genes Dev 2013; 28: 58–70. 10.1101/gad.232009.113 PMC3894413 24395247

[bib42] Soucek L, Evan GI. The ups and downs of Myc biology. Curr Opin Genetics Dev 2010; 20: 91–95. 10.1016/j.gde.2009.11.001 PMC2822095 19962879

[bib43] Evan GI, Wyllie AH, Gilbert CS, Littlewood TD, Land H, Brooks M et al. Induction of apoptosis in fibroblasts by c-myc protein. Cell 1992; 69: 119–128. 1555236 10.1016/0092-8674(92)90123-t

[bib44] Strasser A, Elefanty AG, Harris AW, Cory S. Progenitor tumours from Em-*bcl*-2-*myc* transgenic mice have lymphomyeloid differentiation potential and reveal developmental differences in cell survival. EMBO J 1996; 15: 3823–3834. 8670887 PMC452067

[bib45] Hobeika E, Thiemann S, Storch B, Jumaa H, Nielsen PJ, Pelanda R et al. Testing gene function early in the B cell lineage in mb1-cre mice. Proc Natl Acad Sci USA 2006; 103: 13789–13794. 16940357 10.1073/pnas.0605944103 PMC1564216

[bib46] McCormack MP, Forster A, Drynan L, Pannell R, Rabbitts TH. The LMO2 T-cell oncogene is activated via chromosomal translocations or retroviral insertion during gene therapy but has no mandatory role in normal T-cell development. Mol Cell Biol 2003; 23: 9003–9013. 14645513 10.1128/MCB.23.24.9003-9013.2003 PMC309712

[bib47] Igarashi H, Gregory SC, Yokota T, Sakaguchi N, Kincade PW. Transcription from the RAG1 locus marks the earliest lymphocyte progenitors in bone marrow. Immunity 2002; 17: 117–130. 12196284 10.1016/s1074-7613(02)00366-7

[bib48] Adams JM, Cory S. The Bcl-2 protein family: arbiters of cell survival. Science 1998; 281: 1322–1326. 9735050 10.1126/science.281.5381.1322

[bib49] Schmidt-Supprian M, Rajewsky K. Vagaries of conditional gene targeting. Nat Immunol 2007; 8: 665–668. 17579640 10.1038/ni0707-665

[bib50] Leverson JD, Zhang H, Chen J, Tahir SK, Phillips DC, Xue J et al. Potent and selective small-molecule MCL-1 inhibitors demonstrate on-target cancer cell killing activity as single agents and in combination with ABT-263 (navitoclax). Cell Death Dis 2015; 6: e1590. 25590800 10.1038/cddis.2014.561 PMC4669759

[bib51] Goodwin CM, Rossanese OW, Olejniczak ET, Fesik SW. Myeloid cell leukemia-1 is an important apoptotic survival factor in triple-negative breast cancer. Cell Death Differ 2015; 22: 2098–2106. 26045046 10.1038/cdd.2015.73 PMC4816117

[bib52] Adams JM, Harris AW, Pinkert CA, Corcoran LM, Alexander WS, Cory S et al. The c-*myc* oncogene driven by immunoglobulin enhancers induces lymphoid malignancy in transgenic mice. Nature 1985; 318: 533–538. 3906410 10.1038/318533a0

[bib53] Okamoto T, Coultas L, Metcalf D, van Delft MF, Glaser SP, Takiguchi M et al. Enhanced stability of Mcl1, a prosurvival Bcl2 relative, blunts stress-induced apoptosis, causes male sterility, and promotes tumorigenesis. Proc Natl Acad Sci USA 2014; 111: 261–266. 24363325 10.1073/pnas.1321259110 PMC3890801

